# Demineralized dentin matrix technique - a comparison of different demineralizing solutions

**DOI:** 10.1590/0103-6440202305353

**Published:** 2023-10-27

**Authors:** Fabiano Luiz Heggendorn, Márcio Batista do Nascimento, Andreza Menezes Lima, Alexandre Antunes Ribeiro

**Affiliations:** 1 Postgraduate Program in Dentistry (PPGO) at UNIGRANRIO, Street Prof. José de Souza Herdy, 1,160, block C, 2nd floor - 25th of August - Duque de Caxias - Rio de Janeiro, Brazil. Zip code 25071-202.; 2 Laboratory of Powder Technology, Division of Materials, National Institute of Technology, N° 82 Venezuela Avenue, Room 602, Zip code 20081-312, Rio de Janeiro, RJ, Brazil.

**Keywords:** scaffold, autogenous graft, demineralized dentin matrix, microstructural analysis

## Abstract

This study aimed to evaluate the microstructure formed after the chemical treatment of teeth, for the development of autogenous grafts from the demineralized dentin matrix (DDM) technique, in order to identify the most efficient demineralizing solution. The specimens, originating from the root and coronal portion, were submitted to ultrasonic cleaning and drying in an oven for 1h at 100 ºC. Then, the density was determined by Archimedes’ principle for each specimen, using distilled water as immersion liquid. The samples were separated into five groups: Control group: negative control, Distilled water;EDTA group: positive control, trisodium EDTA; NaOCl group: 2.5% sodium hypochlorite; HCl-0.6M group: 0.6M hydrochloric acid; and H_2_O_2_/H_2_SO_4_ group: hydrogen peroxide and sulfuric acid. Each specimen was immersed for 1h in the corresponding group descaling solution at 60 ºC. Subsequently, the mass loss and density of the treated specimens were determined by Archimedes’ principle. Ultimately, the specimens of each group were characterized by microtomography, Scanning Electron Microscopy, and Energy Dispersive Spectrometry X-ray (SEM-EDS). The results demonstrated that the H_2_O_2_/H_2_SO_4_ solution allowed the formation of interconnected micropores, suggesting better pore structures for application in scaffolds, when compared to the other studied solutions.

## Resumo

Este estudo teve como objetivo avaliar a microestrutura formada após o tratamento químico em dentes, para o desenvolvimento de enxertos autógenos a partir da técnica de matriz de dentina desmineralizada (DDM), a fim de identificar a solução desmineralizante mais eficiente. Os espécimes, provenientes da raiz e porção coronal, foram submetidos à limpeza ultrassônica e secagem em estufa por 1h a 100 ºC. Em seguida, a densidade foi determinada pelo princípio de Arquimedes para cada espécime, utilizando água destilada como líquido de imersão. As amostras foram separadas em cinco grupos: Controle: controle negativo, Água destilada; EDTA: controle positivo, EDTA trissódico; NaOCl: hipoclorito de sódio 2,5%; HCl-0.6M: ácido clorídrico 0,6M; e H_2_O_2_/H_2_SO_4_: peróxido de hidrogênio e ácido sulfúrico. Cada espécime foi imerso por 1h na solução descalcificante de grupo correspondente a 60 ºC. Posteriormente, a perda de massa e a densidade dos espécimes tratados foram determinadas pelo princípio de Arquimedes. Por fim, os espécimes de cada grupo foram caracterizados por microtomografia, microscopia eletrônica de varredura e espectrometria de energia dispersiva de raios-X (SEM-EDS). Os resultados demonstraram que a solução H_2_O_2_/H_2_SO_4_ permitiu a formação de microporos interligados, sugerindo melhores estruturas de poros para aplicação em scaffolds, quando comparada às demais soluções estudadas**.**

## Introduction

Regarding biomaterials, the application of human teeth as a potential biomaterial for bone grafting procedures is little investigated ^1^. Few studies have investigated human dentin from the biomaterial perspective for bone regeneration, choosing to discard extracted human teeth as infectious waste ^1^.

It is worth mentioning the clinical legality of this type of autogenous graft since, in dental practice; root remains are found or even originated from root burials without damage to the patient ^2^. In addition, the federal agency of the United States Department of Health and Human Services (Food and Drug Administration) considers the demineralized dentin matrix (DDM) technique as a reprocessed human tissue and not a medical device, not needing to undergo the rigorous efficacy tests that a medical device requires ^3^.

An autogenous dental bone graft can be a scaffold or a source of growth factors, enhancing bone gain ^4^ ^,^ ^5^ ^,^ ^6^ ^,^ ^7^. Structurally and biochemically similar to bone, DDM demonstrated the presence of exogenous bone morphogenic proteins and other growth factors that promote bone remodeling ^6^.

Different methods are reported in dentin preparation, such as dentin extraction, dentin particles, lyophilized dentin, denatured dentin, and dentin demineralization ^4^ ^,^ ^5^, whereas demineralization is the most popular process in tissue engineering, which can be associated with the use of sonication, vacuum, and temperature ^4^ ^,^ ^8^. Regarding the demineralizing agent, the literature has shown several protocols using, for example, hydrochloric acid (HCl) ^9^, ethylenediaminetetraacetic acid (EDTA) ^10^, and sodium hypochlorite (NaOCl) ^11^. However, no reports were found of using a solution of hydrogen peroxide and sulfuric acid (H_2_O_2_/H_2_SO_4_) as a demineralizing solution.

Bone grafts from autogenous teeth are developed in particulate, powder, and block forms ^2^ ^,^ ^12^ ^,^ ^13^. The block type presents osteoinductive capacity through the blood wettability and osteoconductive, through the maintenance of the space to be regenerated with a slow replacement ^1^ ^,^ ^2^ ^,^ ^12^ ^,^ ^13^. The powder type is studied in different granulations and porosities, having osteoconduction, osteoinduction, and slow replacement ^2^ ^,^ ^12^ ^,^ ^13^. Both are used in isolation or combination in alveolar preservation, aesthetic restoration of alveolar bone, sinus membrane repair, maxillary bone lift, bone crystal augmentation made with guided bone regeneration (GBR), and early implant stability augmentation ^1^ ^,^ ^2^ ^,^ ^12^ ^,^ ^13^.

The block dentin matrix increases surface area for contact with undifferentiated mesenchymal cells compared to the particulate form ^5^. For Catanzaro-Guimarãens et al. ^5^, this statement would respond to greater cytodifferentiation, with consequent bone regeneration of the DDM discs compared to the particulate form.

Studies using the dentin matrix, disc-shaped or particulate, indicated an active process of resorption and replacement in the grafted bone sites ^5^ ^,^ ^6^. The matrix resorption process occurs in parallel with the replacement by the new bone slowly formed. Initially, the fibrocellular tissue surrounding the biomaterial triggers the process of resorption of the dentin matrix surface ^1^ ^,^ ^5^ ^,^ ^6^. The collagenolytic activity ensures the partial internal degradation of the dentin matrix, forming gaps where mesenchymal cells differentiate into osteoblasts ^1^ ^,^ ^5^ ^,^ ^6^. At this point, the matrix is replaced with gradual bone deposition on its surface and inside the biomaterial ^1^ ^,^ ^5^ ^,^ ^6^.

Dentin, after decalcification, can be defined as a matrix composed of type I collagen associated with growth factors ^1^. Cement, a bone-like material, contains transforming growth factor (TGF), insulin-like growth factor type 1 (IGF I), and collagen type I and type III, contributing to osteoinduction ^6^.

Enabling bone induction through decalcified dentin for bone grafts in the mandible would be a crucial milestone in implantology ^4^ ^,^ ^14^. However, few studies have been performed regarding the preparation process, particle size, and shape, which play a critical role in osteoinduction and osteoconduction compared to other biomaterials for osteogenic differentiation ^14^. Therefore, the null hypothesis in this study is that there is no microstructural difference after chemical treatment associated with different solutions of HCl, EDTA, NaOCl, and H_2_O_2_/H_2_SO_4_ for the development of autogenous graft from the demineralized dentin matrix (DDM) technique.

This study aimed to analyze the microstructure formed after the chemical treatment of teeth in solutions of HCl, EDTA, NaOCl, and H_2_O_2_/H_2_SO_4_ for the development of autogenous graft from the demineralized dentin matrix (DDM) technique, in order to identify the best methodology to be used for its production, as well as evaluating the use of the H_2_O_2_/H_2_SO_4_ solution as a potential demineralizing agent.

## Material and methods

The project was submitted to the ethics committee in human research of the University of Grande Rio (UNIGRANRIO) under the number: CAAE 47984521.0.1001.5283.

Eight molar teeth with extraction indication due to orthodontic reasons or accentuated vertical bone loss were used, stored in saline solution, and frozen until the moment of the study. The exclusion criteria adopted concern teeth with coronary restoration, endodontic treatment, and coronary destruction.

### Sample size calculation

The sample calculation determined a minimum number of two dentin disks per condition, for the step of immersion of the dentin matrix in demineralizing solutions, indicated by the Power Analysis or Power Test, difference between 2 Media with Independent Groups through the t Test (http: //calculoamostral.bauru.usp.br/calculoamostral/ta_diferenca_media_independente.php), where a beta error of 20% and an alpha error of 5% were applied. The standard deviation used was ± 2.609, with a difference between the means of 10.244, following a previous study on degradation in DDM by Tanwatana et al. ^15^. However, a sample number of 3 specimens per condition was used.

### Sample preparation

The teeth were manually cleaned with curettes to remove tissue debris, followed by immersion in distilled water in an ultrasonic tank (Elmasonic P 30H, Elma Schmidbauer GmbH, Germany) at 80 Hz for ten minutes at 40 °C.

### Preparation of dentin discs

A diamond disc transversely sectioned the teeth, under refrigeration, in an Isomet cutting machine (Isomet Low Speed Saw, Buehler), obtaining two discs of each tooth, with a thickness between 3 and 4 mm each, one specimen coming from the root area, below the amelocementary line (SP root), and the other from the coronary region (SP crown). From the 16 obtained SP, 15 of them were used for the tests.

A bath in an ultrasonic tank (Maxiclean 750A), previously fed with distilled water heated to 74 ºC, cleaned the specimen again: after being placed in 10 ml penicillin flasks with 5 ml of distilled water, the TBs were taken to an ultrasonic tank at 25 kHz for 20 min at 60 ± 5 °C. Subsequently, distilled water rinsed the samples three times, followed by a drying oven (NT 512, New technique) for one hour at 100 ºC, removing all residual moisture to determine density by Archimedes’ principle and mass loss.

### Determination of density by the Archimedes’ principle

The tests for determining density by Archimedes’ principle are based on ASTM B962-17 ^16^ and follow the steps described below.

### Dry Mass (DM)

Initially, the Dry Mass (DM) values were determined for each specimen, weighed on an analytical balance (Uni Bloc Shimadzu, AUY220) with a kit to determine the density of solids by Archimedes’ principle.

### Apparent mass or submerged mass (AM)

With the specimens immersed in 5 ml of distilled water in 10 ml penicillin flasks, the samples were taken to a heating plate (IKA® Ret Basic) in a water bath with distilled water heated to 100 ºC for 1 hour to fill the pores with water and eliminate the air from the pores and the dentin surface.

The samples remained submerged until complete cooling to 26 ºC (room temperature) when the new weighing was measured on a thousandth scale to obtain the apparent mass measurement (AM) of each specimen. This weighing system consisted of a kit for density determination by Archimedes’ principle submerging the SP in water at room temperature (26 ºC) until the weight stabilized on the scale.

### Wet mass (WM)

Then, the excess water was removed from the surface of the specimens with a cloth moistened with distilled water at room temperature and weighed to obtain the Wet Mass (WM).

The above data establishes the initial parameters, proceeding to the immersion step with the demineralization solutions.

### Calculation of density (()

The density (() of each SP was determined by Eq. 1. The water density value is defined as a function of the temperature measured during the test ((H_2_O at 26 ºC = 0,9967870 g/cm^3^) ^17^.

$$\rho =\;[DW/(WM\;-\;AM)]\;x\;\rho H_{2}O$$ Eq. 1.

### Calculation of relative density (RD)

Relative Density (RD) was determined by Eq. 2, where (_tooth_ = 2.14 g/cm^3^ is the theoretical density of dentin and ρ_enamel_ = 3.0 g/cm^3^ is the theoretical density of the coronary portion samples ^18^.

$$RD\;=\;(\rho_{Sample}/\rho_{tooth})\;x\;100$$ Eq. 2

### Calculation of porosity (Poros.)

The Porosity (Poros.) was determined by Eq. 3.

(Poros.) = 100 - RD Eq. 3.

After obtaining the previous parameters for determining the density by Archimedes’ principle, the specimens remained in an oven for one hour at 100 °C, removing all residual moisture to perform the immersion test in demineralizing solutions for the development of the DDMs.

### Immersion of the dentin matrix in demineralizing solutions

The specimens were immersed in 5 ml of the corresponding descaling solution of their group in 10 ml penicillin flasks, and the set was bathed in ultrasonic bath (Maxiclean 750A) with distilled water preheated at 60 ºC for one hour at 25 kHz. Each group presented three specimens, randomly distributed in the following solutions:

Control group: negative control, distilled water (n: 3);

EDTA group: positive control, trisodium EDTA (n: 3);

NaOCl group: 2.5% sodium hypochlorite (n: 3);

HCl-0.6M group: 0.6M hydrochloric acid (n: 3) and

H_2_O_2_/H_2_SO_4_ (1:1) group: hydrogen peroxide and sulfuric acid (n: 3). H_2_O_2_ and H_2_SO_4_ were mixed in an ice bath to exothermic control. The mixture stood in the ice bath until it reached room temperature (26 °C).

The specimens of EDTA, NaOCl, HCl-0.6M, and H_2_O_2_/H_2_SO_4_ groups were rinsed with 5 ml of distilled water for ten minutes at 25 kHz in an ultrasonic tank at 60 °C. Subsequently, immersed in 5 ml of 99.8% ethyl alcohol, in 10 ml penicillin-type vials, in an ultrasonic tank at 25 kHz for ten minutes at room temperature, followed by drying in an oven for 1 h at 100 °C.

### Post-Decalcification Analysis

### Weight loss

A milesimal precision scale (Bel Engineering) determined the weight of the specimens, making it possible to quantify the weight loss (%) of the DDMs, through the difference between the DM after and the DM before demineralization. The mass loss is determined by Eq. 4.

$$Mass\;loss\;\%\;=\;{DM}_{\left(after\;demineralization\right)}\;\;-\;{DM}_{\left(before\;demineralization\right)}\;/100$$ Eq. 4.

### Density by Archimedes’ principle of demineralized specimens

The demineralized specimens followed a similar protocol to determine the density by Archimedes’ principle, as previously described, obtaining the final density ((_final_), Final Relative Density (RD_final_), and Final Porosity (Poros._final_). From the difference between the final data (after immersing in descaling solutions) and the initial data (before immersing in descaling solutions), the resulting density ((_resultant_), Relative Density (RD_resultant_), and Porosity (Poros._resultant_) were determined.

### Microstructural and morphological analysis

Subsequently, Scanning Electron Microscopy (SEM, QuantaT^M^ 450 FEG, Oxford Instruments) and elementary microanalysis by Energy Dispersive Spectrometry X-ray (SEM-EDS) analyzed the microstructure and morphology of two specimens from each group. All samples were coated with a thin film of gold (Au) under an argon atmosphere, using the sputtering process (Emitech SC7620 Sputter Coater), to make them conductive. We used magnification of 500x, 1000x, 2500x, 5000x, 10000x and 20000x. In addition, the opening diameter of the dentinal tubules in the different groups was measured by the calibrated scale bar of the SEM-FEG microscope imaging software (FEI Company - xTM version 4.1.11.2145), using one image of each group with 5000x magnification.

### 3D Micromorphological Analysis

A SkyScan 1172 microtomography (µCt) (Bruker-μCT, Kontich, Belgium) analyzed all the specimens before and after the density determination test by Archimedes’ principle (EDdmA), with the following parameters: voltage 50 Kvp; Source Current 800 μA, flat-field correction, Al filter 0.5, Image Pixel size 21.98 μm; Exposure 4000 ms; Rotation Step 0.5, Frame Averaging 3.

The NRecon program (SkyScan, Kontich, Belgium) reconstructed the images obtained from μCT, adjusting the parameters: Ring artifacts reduction 7, Beam-hardening correction 46%, Smoothing Kernel Gaussian, Defect pixel masking 50%, and Misalignment Compensation. Subsequently, the CTan program (SkyScan, Kontich, Belgium) segmented the images, delimiting the regions of interest (ROI) of the samples. The ROI of each sample followed the process of thresholding and binarization of the images, adjusting the histogram to show the porosity in the samples. Ultimately, 3D images were visually evaluated in DataViewer and CtVox software (SkyScan, Kontich, Belgium).

The analyses of structure and architecture followed the morphometric parameters: percentage of bone volume (BV/TV%), total pore volume (Po.V(tot)%), and total porosity (Po(tot)%). The quantification of dentin demineralization achieved in each group is determined between the parameters obtained after immersion of the demineralized dentin matrix subtracted by the value of the corresponding parameter obtained in the initial condition of each specimen.

### Statistical analysis

In the GraphPad Prisma 5.01 software (Graph Pad Software Inc), the data were analyzed using the analysis of variance method (One-Way - ANOVA) and complemented by the Tukey post-test, with a significance level of 5% (p < 0.05).

## Results

### Weight loss

**Figure 1:**
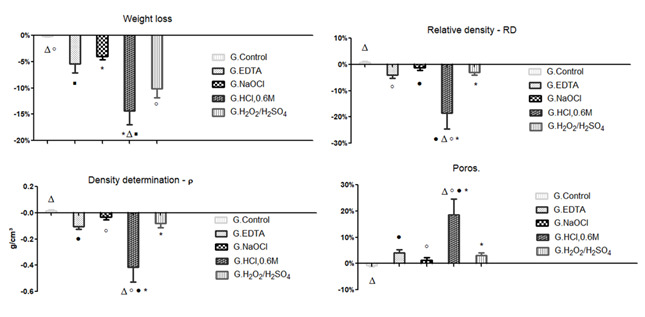
Average mass loss and determination of the final density, the final relative density, and the final pores after the demineralization tests.

The analysis revealed a greater mass loss in HCl-0.6M group (-14.39 ± 4.50%), followed by H_2_O_2_/H_2_SO_4_ (-10.12 ± 2.94%), EDTA (-5.41 ± 2.98%), NaOCl (-4.04 ± 1.05%) and Control groups (-0.20 ± 0.17%). Comparing mass loss between groups revealed a statistical difference between:Control and HCl-0.6M groups; Control and H_2_O_2_/H_2_SO_4_ groups; EDTA and HCl-0.6M group; NaOCl and HCl-0.6M group (Figure.1).

The slight mass loss observed in Control group (-0.20 ± 0.17%) suggested an effect of the ultrasonic bath, performed in the cleaning step, leading to the detachment of material in the SPs or the removal of residual impurities.

### Density determination by the Archimedes’ principle

The difference between the final and initial values of ( and RD showed a greater reduction in HCl-0.6M group ((_resultant_ = -0.42 ± 0.19 g/cm^3^; RD_resultant_ = -18.45 ± 10.58%), EDTA group ((_resultant_ = -0.11 ± 0.03 g/cm^3^; RD_resultant_ = -4.10 ± 1.94%), H_2_O_2_/H_2_SO_4_ group ((_resultant_ = -0.08 ± 0.06 g/cm^3^; RD_resultant_ = -3.10 ± 1.65%) and NaOCl group ((_resultant_ = -0.03 ± 0.04 g/cm^3^; RD_resultant_ = -1.31 ± 1.55%), while in Control group ((_resultant_= 0.01 ± 0.01 g/cm^3^; RD_resultant_ = 0.65 ± 0.74%) the density remained stable. The porosity difference (Poros.) also followed the same pattern as the groups above, revealing an increase in HCl-0.6M (Poros._resultant_ = 18.45 ± 10.58%), EDTA (Poros._resultant_ = 4.10 ± 1.94%), H_2_O_2_/H_2_SO_4_ (Poros._resultan t_= 3.10 ± 1.65%), and NaOCl groups (Poros._resultant_ = 1.31 ± 1.55%), while in Control group (Poros._resultant_ = -0.65 ± 0.74%) the porosity remained stable.

The slight variation in the values of (, RD and Poros. of the control group was not significant and can be considered an intrinsic error to the method, which may be associated with the operator, the sensitivity of the analytical balance, or the hydration of the samples.

The comparison of the data between the groups, revealed a statistical difference between: Control and HCl-0.6M groups; HCl-0.6M and H_2_O_2_/H_2_SO_4_ groups; EDTA and HCl-0.6M groups; NaOCl and HCl-0.6M groups (Figure. 1).

Microstructural and morphological analysis

For each group, the SPs crown were analyzed in the enamel and dentin regions, while the SPs root scans occurred in the dentin portion, whereas two scans analyzed each specimen.

The SEM images revealed morphological differences in the enamel and dentin surface between the groups.

**Figure 2:**
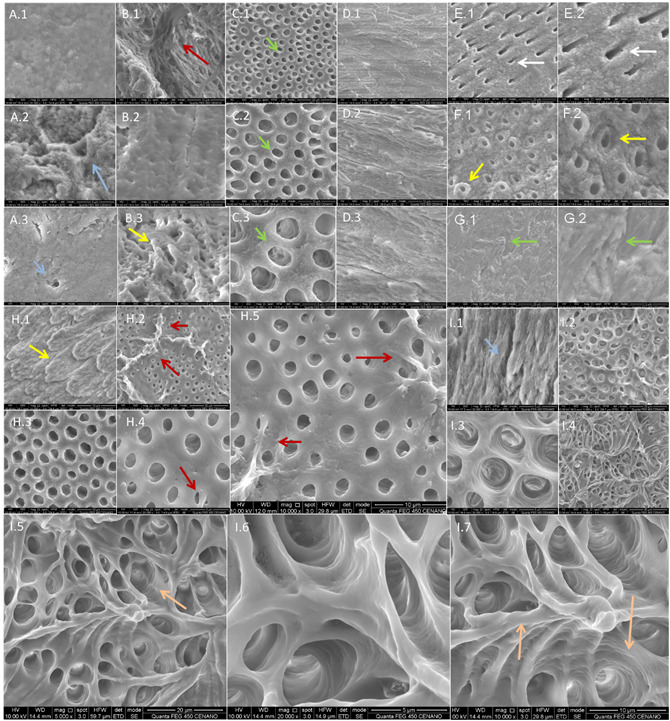
SEM images of Control and EDTA groups. Micrographs of Control group showing in the enamel a rough surface with debris (A.1) and in dentin, dentinal tubules partially obstructed by smear layer (A.2 and A.3, blue arrows). Photomicrographs of the EDTA group suggest the presence of collagen fibers (B.1, red arrow), smear layer surface layer (B.2), and projections on the surface (B.3, yellow arrow). CP crown of the EDTA group revealing unobstructed dentinal tubules (C.1 to C.3, green arrows), while in the enamel region is possible to identify an irregular and rough surface (D.1 to D.3). Images of the SP root from group NaOCl reveal open dentinal tubules (E.1 and E.2, white arrows), while the SP crown from group NaOCl shows a surface with exposed dentinal tubules surrounded by a projected peritubular region (F.1 and F.2, yellow arrows). The enamel region demonstrates areas suggestive of overlapping lamellae (G.1 and G.2, green arrows). Micrographs of theHCl-0.6M group demonstrated in the enamel layer a layered lamellar surface (H.1, yellow arrow), and in dentin deposits suggestive of collagen fibers (H.2, H.4 and H.5, red arrows), while other regions presented a free surface, with a total exposure of the dentinal tubules (H.3). The H_2_O_2_/H_2_SO_4_ group shows areas of enamel loss (I1, blue arrow) and in the dentin region, an interconnected morphology, composed of decalcified dentin (I2 to I7) with dilation of the region of the dentin tubules and deep demineralized areas (I.5 and I.7, orange arrows).

In Control group, images A.1, A.2, and A.3 of Figure. 2suggested the presence of an intense smear layer, leaving obliterated the mouth of the dentinal canaliculi of the SP crown, while in the SP root, a clogged dentinal canaliculus can be visualized (Figure. 2 A.3). The surfaces presented a rough and irregular morphology, with debris (Figure. 2A.1 A.2 and A.3). The images of EDTA group, from the SP crown, suggested a lower intensity of smear layer and unobstructed dentinal tubules *(* Figure. 2C.1, C.2 and C.3) when compared with those of Control group (Figure. 2A.1 and A.2). The SP root displayed an irregular surface covered with projections, with partially obliterated dentinal tubules (Figure. 2B.1, B.2 and B.3), suggesting the presence of remaining collagen bundles (Figure. 2B.1). In the enamel region, it was possible to identify an irregular and rough surface (Figure. 2D.1, D.2 and D.3).

On the other hand, the NaOCl group presented a surface with a smaller amount of smear layer (Figure. 22 E.1 and E.2), revealing dentinal tubules along the entire surface of the root section (Figure. 2E.1 and E.2) and coronary (Figure. 2F.1, F.2, G.1 and G.2). The dentin presented a decalcification with peritubular halos, bordering the mouths, with an irregular surface in the intertubular dentin. Projections of the dentinal tubules, protected by this peritubular region, are also present. The region of dental enamel had an irregular surface (Figure. 2G.1 and G.2), formed by areas suggestive of lamellae overlapping and smear-layer free.

Among the groups analyzed, HCl-0.6M (Figure. 2H.1-H.5) and H_2_O_2_/H_2_SO_4_ groups (Figure. 2I.1-I.7) presented the highest demineralization. In both groups, the presence of a smear layer on the surface was not identified, the dentinal tubules were completely exposed, and the enamel layer showed an intense demineralization of overlays, such as overlapping lamellae (Figure. 2 H.1 and I.1). In HCl-0.6M group, areas with interconnected beams identified on the surface of the specimen suggest collagen (Figure. 2H.2). The H_2_O_2_/H_2_SO_4_ group (Figure. 2I.2-I.7) revealed an intense demineralization on the surface compared to the other groups, revealing exposed dentinal tubules, below the level of the dentinal surface, with intertubular demineralization. The images of H_2_O_2_/H_2_SO_4_ group (Figure. 2I.5-I.7) resembled scaffolds, having an intense inter- and intratubular porosity, suggesting a decalcifying action on the group in depth, forming artificial channels due to porosity (Figure. 2I.5-I.7). The dentin region (Figure. 2I.5-I.7) demonstrated an interconnected morphology composed of a decalcified dentin, similar to graft scaffolds, with dentin bridges interconnecting internal areas of the specimens. An evident dilation of the dentinal tubule region suggests the formation of deep demineralized areas (Figure. 2I5-I.7).

The microstructural and morphological analysis are complemented by measurements of the diameters of the dentinal tubule mouths, in the different groups, at a magnification of 5000x. For this analysis, a variable quantity of opening diameters (QOD) was obtained by assessing only one SEM image of each group, and the QOD number for each group was described. Measurements only in one field of each specimen revealed the largest diameters of the dentinal tubules in group H_2_O_2_/H_2_SO_4_, root section (mean: 3.82 ± 0.98 µm) (QOD n: 11) and coronary (mean: 3.54 ± 0.99 µm) (QOD n: 24), followed by groups EDTA, coronary section (mean: 2.81 ± 0.52 µm) (QOD n: 20); HCl-0.6M, coronary section (mean: 2.61 ± 0.35 µm) (QOD n: 32); NaOCl, root section (mean: 1.87 ± 0.34 µm) (QOD n: 16); HCl-0.6M, root section (mean: 1.83 ± 0.46 µm) (QOD n: 32); NaOCl, coronary section (mean: 1.27 ± 0.19 µm) (QOD n: 26) and Control, root section (mean: 1.24 ± 0.50 µm) (QOD n: 7).

### X-Ray Dispersive Energy Spectrometry

**Figure 3:**
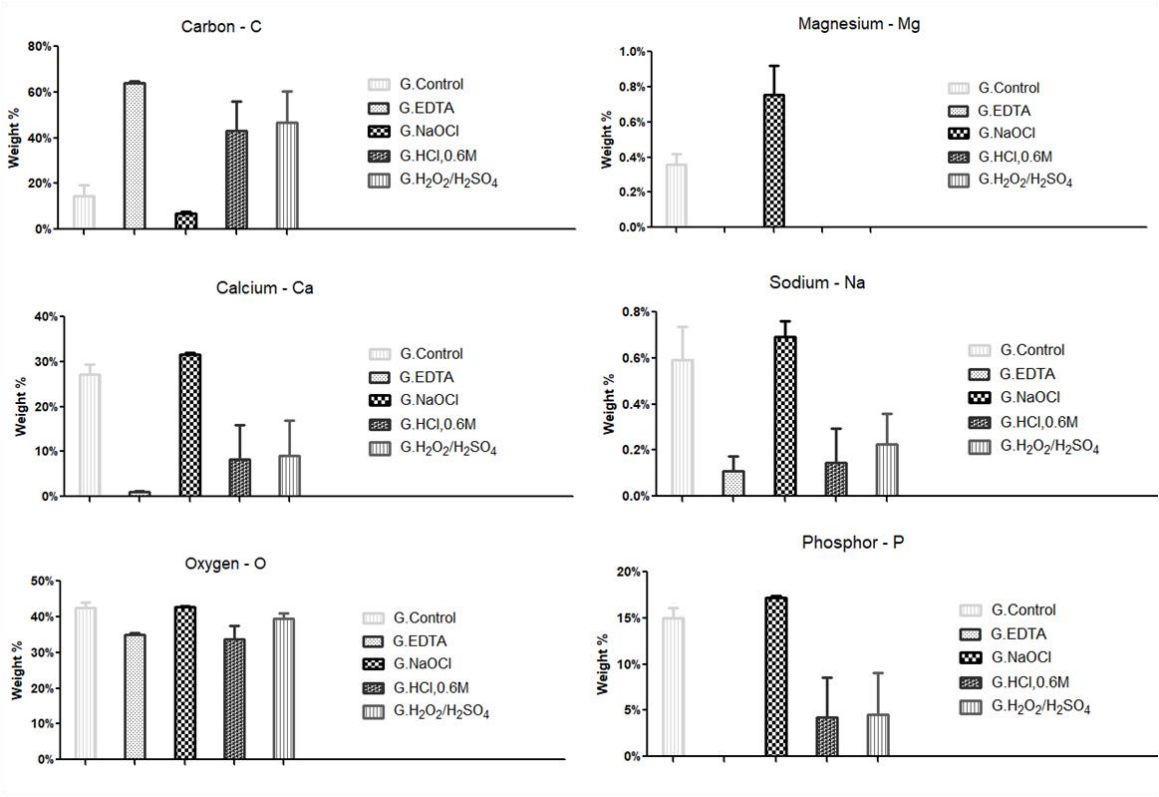
Chemical analysis by energy dispersive spectrometry of X-rays. Analysis of the spectra identified on the surfaces of the groups after the action of the different demineralizing solutions.

Corroborating the analysis of the SEM images, the spectra revealed in SEM-EDS indicated the lowest concentrations of phosphorus (P) and calcium (Ca) in the EDTA groups, followed by H_2_O_2_/H_2_SO_4_ and HCl-0.6M groups (Figure. 3), suggesting greater demineralizing potential of EDTA and H_2_O_2_/H_2_SO_4_ groups, followed by HCl-0.6M group. These same groups had the lowest levels of sodium (Na) and oxygen (O), while NaOCl group had the highest levels of these elements, which the permanence of residual elements of NaOCl on the DDM can partially explain. In addition, magnesium (Mg) was not identified in the EDTA, HCl-0.6M, and H_2_O_2_/H_2_SO_4_ groups, while Control and NaOCl groups presented high percentages of this element. The increased level of Mg content in the NaOCl groups is in accordance with the literature, which has reported that NaOCl is able to expose the inorganic material that prevents further dissolution of the dentin or it may dissolve the organic components and leave a smear layer of mineralized tissue (19, 20 , 21).

The high loss of P, Ca, Mg, and Na, in association, with the groups EDTA, H_2_O_2_/H_2_SO_4,_ and HCl-0.6M, enabled the formation of matrices composed of high levels of Carbon (C) (Figure. 3). The significantly elevated presence of C in groups EDTA, H_2_O_2_/H_2_SO_4_, and HCl-0.6M suggests a decalcified matrix. However, the analysis of G in the H_2_O_2_/H_2_SO_4_ and HCl-0.6M groups showed a diversified composition of O, C, P, and Ca, with reduced levels of Na. In contrast, the composition of the DDM processed in the EDTA group was limited to a matrix of O and C, with reduced levels of Ca and Na.

### 3D Micromorphological Analysis

**Figure 4:**
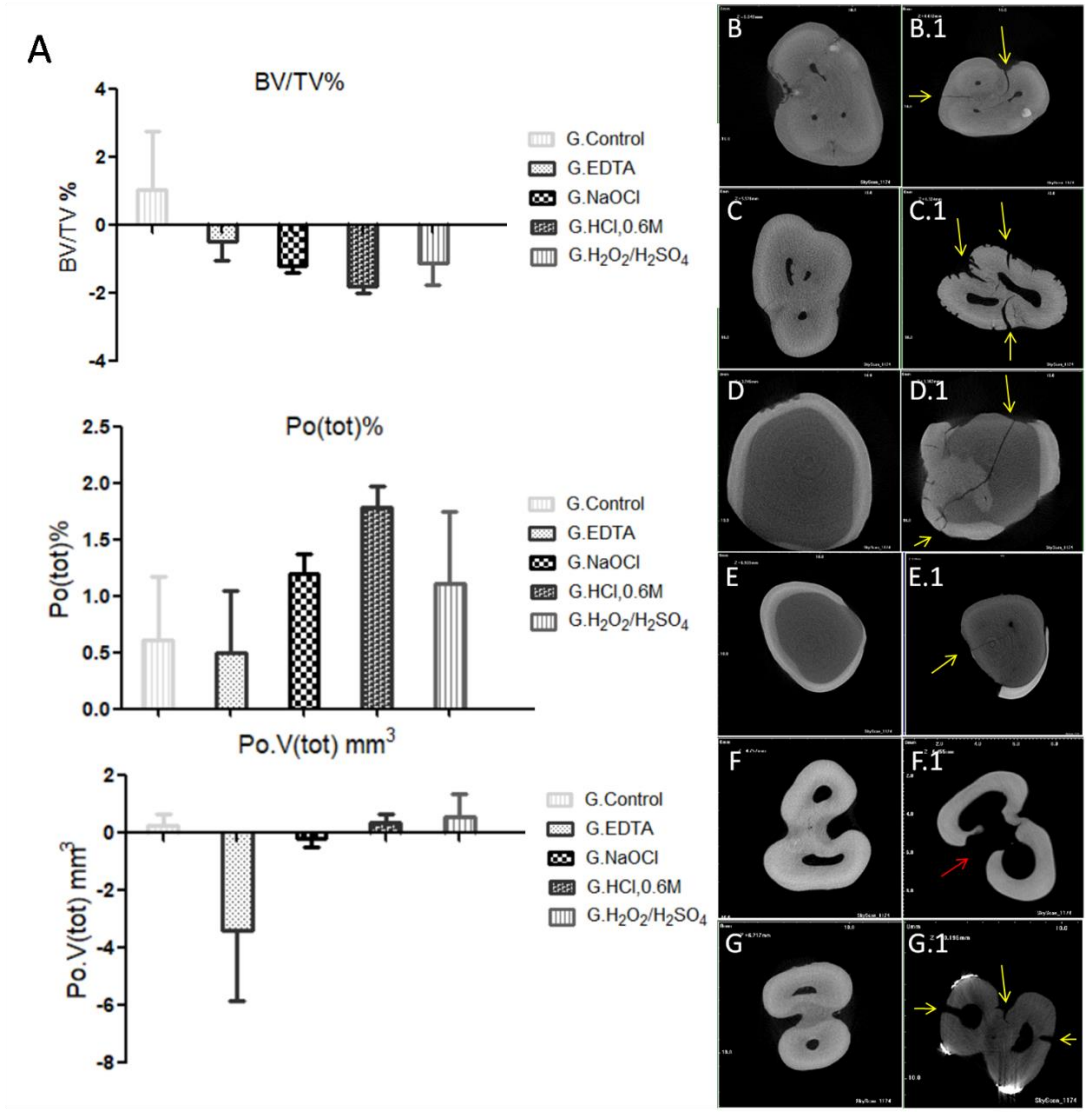
3D Micromorphological Analysis. Statistical graphs of BV/TV%, Po(tot)%, and Po.V(tot)% parameters (A), resulting after EDdmA. Analysis of 3D reconstruction in the DataViewer program revealing cracks after EDdmA (yellow arrows), with lower intensity in Control, NaOCl, and EDTA groups after EDdmA (B.1, E.1 and D.1), when compared with the previous condition (B, E, and D), respectively. The H_2_O_2_/H_2_SO_4_ group after EDdmA (C.1 and G.1), presented a higher number of cracks and fissures when compared to the CPs before EDdmA (C and G). The HCl-0.6M group after EDdmA (F.1) showed a substantial loss of dentin on the external face and in the intraradicular region, communicating these regions (red arrow) when compared to the condition before the EDdmA (F).

The analysis revealed no statistical difference in the parameters BV/TV% (p = 0.273), Po.V(tot)% (p = 0.1822), and Po(tot)% (p = 0.3548) when comparing the groups of demineralized dentin matrices. However, BV/TV% showed a greater reduction in the ratio of trabeculae in the HCl-0.6M group (-1.795 ± 0.325%), followed by NaOCl (-1.199 ± 0.314%), H_2_O_2_/H_2_SO_4_ (-1.119 ± 1.094% ) and EDTA groups (-0.189 ± 0.493%). The Control group also presented an increase in BV/TV% (+1,356 ± 2,851%) (Figure. 4).

The ratio of the total pore volume (Po.V(tot)%), calculated as the volume of all open and closed pores as a percentage of the total volume of interest, showed the highest Po.V(tot)% in H_2_O_2_/H_2_SO_4_ (+0.545 ± 1.395%), HCl-0.6M (+0.343 ± 0.537%), and Control groups (+0.259 ± 0.677%). The groups NaOCl (-0.172 ± 0.568%) and EDTA (-3.406 ± 4.270%) presented a reduction in Po.V(tot)% (Figure. 4).

The total porosity (Po(tot)%) made it possible to determine the porosity gain of the SPs in different conditions. The highest Po(tot)% was determined in HCl-0.6M group (+1.795± 0.325%), followed by NaOCl (+1.199 ± 0.314%), H_2_O_2_/H_2_SO_4_ (+1.119 ± 1.094%), Control (+0.613 ± 0.977%) and EDTA groups (+0.495 ± 0.958%) (Figure. 4).

Micromorphological analysis of 3D reconstruction in the DataViewer program revealed the presence of cracks after EDdmA (yellow arrows), with less intensity, in isolation, in Control, NaOCl, and EDTA groups after EDdmA (Figure. 4B.1, E.1 and D.1), when compared to the previous condition (Figure. 4B, E, and D), respectively. H_2_O_2_/H_2_SO_4_ group after EDdmA (Figure. 4C.1 and G.1) presented a greater number of cracks and fissures of different sizes along the external face of the specimens when compared to the specimens before EDdmA (Figure. 4C and G). An increase in the diameter of the areas corresponding to the intraradicular region also occurred, indicating demineralization. The HCl-0.6M group after EDdmA (Figure. 4F.1) presented a strong loss of dentin matrix on the external face and in the intraradicular region, communicating these regions (red arrow) when compared to the condition before the EDdmA (Figure. 4F). It was also possible to visualize in the Control group the presence of cracks after EDdmA, in lesser intensity, penetrability, and size, when compared to the other groups after EDdmA. The presence of these artifacts, such as reduced cracks in the Control group, can be correlated to sample preparation, such as the possible effect of ultrasound baths and exposure to heat, when in a bath or in an oven, before the immersion tests.

## Discussion

Tissue engineering aims to repair or regenerate lost or damaged tissues by combining scaffolds of biomaterials, cells, and growth factors ^22^. Some biomaterials have a matrix role, creating optimal microenvironmental conditions for local cells as a framework to guide tissue reorganization ^22^. Scaffolds will serve as a transient system, being replaced by local tissue, over time, by degradation or reabsorption ^7^. In addition, biomaterials can serve as vehicles for transporting or releasing bioactive factors in a controlled manner by incorporating bioactive agents, such as proteins and growth factors, thus allowing the adhesion and proliferation of cells with subsequent formation of extracellular matrix ^22^.

The limited availability of allografts and autografts highlights the need to develop new concepts for developing alternative tissues to correct tissue defects ^7^. To expand new treatment strategies for bone gain, different articles have indicated the importance of DDM as an osteoinductive implant material, presenting advantages such as cost/benefit ratio, osteoinductive potential, and chemotactic properties and promoting the acceleration of the bone repair process in surgical bone defects produced ^1^ ^,^ ^5^ ^,^ ^6^.

The methodology for preparing the teeth on dentin discs allowed the exposure of the internal dentin of the root and crown specimens, on the upper and lower face of each disc, maintaining a ratio of predominance of the dentin surface area to the chemical action of the different demineralizing solutions, allowing the enamel, from the coronal portion, to remain around the crown specimen for microstructural and morphological analyses. For the determination of mass loss, this ratio of exposure area/chemical reaction becomes crucial to determine the best demineralizing action, which makes it possible to group the root and crown specimens for analysis. For the determination of density by the Archimedes principle, this relationship was compensated by applying the theoretical density of the dentin and the density of the enamel, making it possible to group the root and crown specimens in the analysis.

### Determination of density by the Archimedes’ principle

Mechanical strength and biological functionality are influenced by the characteristic architectures of scaffolds, including pore size, porosity, interconnectivity, and surface area/volume ratio ^23^ ^,^ ^24^. High porosity is one of the necessary characteristics for uniform cellularity, enabling tissue fixation and neoformation, and acting as artificial vessels in the transmission of molecules and cells ^7^ ^,^ ^24^. An ideal porosity would be 90%, allowing diffusive transport of the cells within the scaffolds ^24^. However, such a high porosity could compromise the mechanical properties of this scaffold ^24^. Table 1 displays the average porosity values calculated from Eq. 3, for each studied group, where it is observed that the G.HCl-0.6M was the one that presented the highest average final porosity.

The average porosity data found for the DDMs from the G.HCl-0.6M group are comparable to the porosities of metal scaffolds developed by 3D printers, ranging from 40% to 70% ^23^. In addition, the literature has reported porosity between 5% and 10% for cortical bone ^7^, which is below the final average porosity of all studied samples. The best framework for DDMs would be supported by the work of Limmahakhun & Yan ^23^, when they conceptualized the “grading of bone scaffolds” developed by 3D or conventional additive methods. In this concept, a functionally graded structure, having distinct porosities, is correlated to a specific space gradient in the scaffold ^23^. This architecture would be superior to others as it favors the necessary physical and mechanical control in areas under physiological stress ^23^. Therefore, functionally graded biomaterials could provide mechanical resistance for implants to withstand physiological loading, while graded porosity optimizes the response of the biomaterial to external loading and allows the formation of slightly larger amounts of bone when compared to scaffolds with homogeneous porosity ^23^.

Such data demonstrate that the methodology for demineralizing DDMs should be improved to achieve greater porosity, thus enabling the development of an efficient biomaterial through the DDM technique. However, the DDMs would not be traditional scaffolds composed of hydrogels, polymers, bioceramics, or laboratory-treated hydroxyapatites. The vast majority of articles that addressed porosity focus on these biomaterials ^7^ ^,^ ^24^, and even with the ideal parameters, after the replacement period, they tend to fracture due to their fragility ^23^.

SEM analyses

For surgical reconstruction, a porous scaffold with an open porosity facilitates the circulation of biological fluids, enabling cellular fixation for tissue neoformation ^7^ ^,^ ^23^. The cellular and regenerative responses of the tissue depend on different characteristics of the pores, such as quantity, shape, size, and interconnectivity ^7^ ^,^ ^23^. Studies have reported that interconnected macropores (> 100 μm) in the range of 200 to 600 μm are the prerequisites for bone growth, cell infiltration, nutrient/excretion transport, and promotion of capillary endogenesis to support bone tissue formation, and micro-pores in the range of 0.5 to 20 μm favor cell adhesion and differentiation ^25^ ^,^ ^26^.

Based on these studies, the samples from all groups studied showed micropore sizes (0,74 to 4,80 µm) favorable to cell adhesion and differentiation. However, the DDMs developed from the EDTA (Figure. 2 C.1-C.3), HCl-0.6M (Figure. 2 H.2-H.5), and H_2_O_2_/H_2_SO_4_ groups (Figure. 2 I.2-I.7) showed more suitable microporosity characteristics in terms of micropore depth to be designed as scaffolds. The samples from the EDTA (Figure. 2C.1-C.3) and HCl-0.6M (Figure. 2 H.2-H.5) groups showed similarity in terms of pore morphology that is closed and rounded pores. The samples from G.H_2_O_2_/H_2_SO_4_ (Figure. 2 I2-I7) showed interconnected micropores, with parts of the peritubular dentin on the dentin surface, suggesting better pore structures for application in scaffolds than the samples from the EDTA (Figure. 2 C.1-C.3) and HCl-0.6M (Figure. 2 H.2-H.5*)* groups. Therefore, the H_2_O_2_/H_2_SO_4_ solution promoted dentin disorganization and interconnectivity between dentinal tubules, suggesting the formation of pores, while EDTA and HCl-0.6M solutions were only able to remove the smear layer and clean the dentinal tubules.

Murphy et al*.* ^25^ indicated that mean pores of 164 µm to 190 µm demonstrated a cell migration with slow progression, not reaching the center of the scaffold, while pores of 325 µm showed a higher rate of cell infiltration associated with uniform cell distribution. In contrast, higher levels of cells have been found in scaffolds with 96 µm pores, indicating that cell adhesion decreases with increasing pore size ^27^. Therefore, the lower availability of specific surface area due to the increase in pore size can reduce cell adhesion areas ^27^.

Considering the data above, roughness and topography characteristics also altered cellular behavior ^28^. Calore et al. ^28^ and Abdollahiyan et al. ^7^ indicated that in an osteogenic environment, human mesenchymal stromal cells and osteoblasts are more responsive to roughness than to surface stiffness of different scaffolds. Correlating this information with the images obtained in the SEM, H_2_O_2_/H_2_SO_4_ group exhibited the best topographic profile, with irregular morphology and intense inter- and intratubular porosity, showing better characteristics than the other groups since Ho & Hutmacher ^24^ indicated the efficiency in cell diffusion in scaffolds with interconnected pores.

The EDS analyses (Figure. 3) showed that the final chemical composition of the sample groups is compatible with the normal bone chemical structure. Abdollahiyan et al. ^7^ reported bone constitution being hydroxyapatite crystals - HA (phosphorus and calcium), chlorine, sodium, magnesium, carbonate, fluorine, potassium, and traces of strontium, zinc, silicon, and copper, in addition to collagen fibers. The presence of Ca and P in H_2_O_2_/H_2_SO_4_ and HCl-0.6M groups, after the process of making the DDMs, suggested findings as promising related to the EDTA group, which presented lower levels of Ca and nulls of P since the presence of hydroxyapatite, composed mainly of Ca and P, increases the mechanical properties and enhances the adhesion capacity of osteoblasts in scaffolds ^7^. Previously, Kuntze et al. ^29^ identified, through EDS, similar Ca and P patterns in demineralized samples with 6 M and 12 M HCl, demonstrating higher percentages of Ca and P, corroborating the findings reported in this study.

The micromorphological analysis corroborated with the mass loss test, (, and DR, maintaining HCl-0.6M and H_2_O_2_/H_2_SO_4_ between the groups with greater loss of BV/TV%, and increase of Po.V(tot)% and Po(tot)%. On the other hand, the Control group, with a gain of BV/TV%, is related to the mass loss test, where it presented a minimum mass loss (-0.20 ± 0.17%). The Po.V(tot)% analysis corroborated with the microstructural and morphological analysis performed in the SEM. The applied PoV analysis (tot)% calculates the volume of all open and closed pores as a percentage of the total volume of interest, which may correspond to a greater number of obliterated tubules, partially or totally, as observed in the SEM of NaOCl, EDTA, and Control groups.

Through the use of density determination by Archimedes’ principle, the best demineralizing potentials are identified in the solutions of HCl-0.6M, H_2_O_2_/H_2_SO_4,_ and EDTA, resulting in microporosity characteristics more suitable in terms of micropore depth to be designed as scaffolds.

The presence of a matrix composed of Ca and P in the groups H_2_O_2_/H_2_SO_4_ and HCl-0.6M, after the process of making the DDMs, allowed us to suggest the feasibility of employing DDM scaffolds in the technique of guided bone regeneration by presenting a mineral profile similar to the bone.

Deepening the analyses, we concluded that the H_2_O_2_/H_2_SO_4_ solution allowed the formation of interconnected micro-pores, suggesting better pore structures for application in scaffolds when compared to the other solutions studied. Even not reaching the highest demineralizing potential, the H_2_O_2_/H_2_SO_4_ solution allowed the development of scaffolds with different chemical and surface characteristics of microporosity, surpassing the other solutions used in the development of DDMs, being determined as the best solution for use in the DDM technique.

Further studies are necessary to develop the DDM technique to correlate the osteogenic potential and with the microstructure.
